# Expression of the apoptosis-related genes BCL-2 and BAD in human breast carcinoma and their associated relationship with chemosensitivity

**DOI:** 10.1186/1756-9966-29-107

**Published:** 2010-08-07

**Authors:** Bing Yu, Xin Sun, Hong-yan Shen, Feng Gao, Yuan-ming Fan, Zhi-jun Sun

**Affiliations:** 1 Department of Breast and Thyroid Surgery, The Second Affiliated Hospital, Chongqing Medical University, 74th Linjiang Road, Yuzhong District, Chongqing, 400010, China

## Abstract

**Objective:**

To evaluate the expression of BCL-2 and BAD genes in tissues of breast carcinoma and investigate the relationship between the expression of BCL-2 and BAD in breast cancer cells with chemosensitivity.

**Methods:**

Immunohistochemical technique was used to detect the expression of BCL-2, BAD in 10 normal breast tissues, 10 breast fibroadenoma tissues, 40 youth human breast carcinoma tissues, 40 menopause human breast carcinoma tissues. And to detect the expression of ER, PR in 80 human breast carcinoma tissues. 20 Surgical samples of breast cancer, diagnosed by pathology, were obtained from The First Affiliated Hospital of Chongqing Medical University. The cancer sample cells were cultured separately in the incubator at 37°C, 5% CO_2 _in vitro. The rate of inhibition of cancer cells in 4 kinds of anticancer drugs-- Epirubicin Adriamycin (EADM),5-Fluorouracil (5-Fu), Navelbine(NVB) and Diaminedichloroplatinum (DDP), were assayed by MTT method.

**Results:**

The expression of BCL-2, BAD genes in young human breast carcinoma tissues were lower than that in menopause human breast carcinoma tissues (***P *< 0.05**). There was a negative correlation between the positive expression rate of BCL-2 and histologic grade or the lymph node metastasis (***P *< 0.05**). There was a positive correlation between the expression rates of BCL-2 and of ER, PR (***P *< 0.05**). The expression of BAD had no relationship with the expression of ER, PR, histologic grade and the lymph node metastasis(***P *= *NS***). Sensitivity rates of 20 breast cancer cells in 0.1 × PPC within 48 h in vitro were 30% EADM,20% 5-Fu,45% NVB and 25% DDP. Respectively, the rate of inhibition of EADM,5- Fu, NVB and DDP were significantly higher in the BCL-2 negative cancer cells than in the BCL-2 positive cancer cells. A negative correlation was found between expression of BCL-2 and chemosensitivity for all the 4 anticancer drugs. The inhibition rates of EADM and NVB were significantly lower in the BAD negative cancer cells than in the BAD positive cancer cells. A positive correlation was found between expression of BAD and chemosensitivity for Epirubicin.

**Conclusion:**

The expression of BCL-2 and BAD can be used as prognosis factors of breast cancer. Detection of the BCL-2 protein expression level, particularly, combined with the detection of the expression of BCL-2 and BAD as well as ER and PR were helpful in confirming the prognosis of breast carcinoma. The combined detection of BCL-2 and BAD may be markers for predicting the responses to anticancer drugs.

## Background

Breast carcinoma is endangering the health of women, its development process involves decreasing expression of apoptosis gene. BCL-2 is a anti-apoptosis gene, the function of BAD gene is promoting the apoptosis of cell. The balance between BCL-2 and BAD can effect the apoptosis of cancer cell. In our study, immunohistochemistry was used to detect the expression of BCL-2 and BAD in breast carcinoma, in addition, to analyze the relationship between the expression of the two genes and the expression of ER, PR histologic grade, clinical stage and the lymph node metastasis.

Chemotherapy is an important therapy to breast cancer. Although there have been introduced new chemotherapeutic agents and new chemotherapy, the effect of chemotherapy in breast cancer is not ideal. An important reason for this is that breast cancer cells to chemotherapeutic agents are neither sensitive nor resistant. Currently looking for the target which could forecast the effect of chemotherapy on breast cancer are largely needed. The EADM, 5-Fu, NVB, DDP are the widely-used first-line chemotherapy drugs for breast cancer in the world. In this study MTT assay was used to analyze the relative inhibition effect of four kinds of chemotherapy drugs which include EADM, 5-Fu, NVB and DDP on breast cancer cells, and the relationship between the expression of BCL-2, BAD and the chemosensitivity.

## Materials and methods

### Materials

1.1.1 We collected 80 samples of breast carcinoma during 1998-2002, originated from The First Affiliated Hospital of Chongqing Medical University. Including 40 youth breast carcinoma tissuses(age < 35 years old), 40 menopause breast carcinoma tissuses(age > 60 years old);11 cases of clinical Stage I, 47 cases of clinical stage II, 19 patients with clinical stage III, 3 patients with clinical stage IV; histological grade I of 26 cases, 46 cases of grade II, III is 8 cases. 10 samples from patients of breast fibroadenoma.10 normal breast tissue samples from 10 patients of side tissue of fibroadenoma. All the samples were made into 5 μm tissue sections

1.1.2 We collected 20 fresh samples of breast cancer, which diagnosed by pathology, without preoperative radiotherapy and chemotherapy, originated from The First Affiliated Hospital of Chongqing Medical University. We selected the samples according to the asepsis operation and avoid the necrosis region. One part of the tumor specimens was resected from the primary lesions and transported to our laboratory as quick as possible in RPMI 1640. The other part was put in formal in fixation, dehydration and paraffin imbedding.

### 1..1.3 Reagent

Rabbit anti-human multiclonal BCL-2 antibody and Rabbit anti-human multiclonal Bad antibody were purchased from Boshide Bio-engineering company. Epirubicin, Fluorouracil, Navelbine and Cisplatin were dissolved in the mother liquor separately by physiological saline, and then disposed the mother liquor into fluid (100 × PPC), positive pressure filtration sterilization, -20°C preservation.

#### 1.2.1 Immunohistochemistry

Immunohistochemistry was carried out on 5 μm tissue sections from paraffin blocks using the avidin-biotin immunoperoxidase method, The following antibodies were used: Rabbit anti-human multiclonal BCL-2 antibody and Rabbit anti-human multiclonal Bad antibody. Briefly, the paraffin sections were deparaffinized with xylene and rehydrated through a series of descending graded ethanol. Endogenous peroxidase activity was blocked by incubation for 15 min in 0.3% H_2_O_2 _buffer. To unmask the epitopes of BCL-2 and BAD microwave-processing pretreatment was carried out in a citrate buffer, pH = 6.0 for 10 min.. Subsequently, Rabbit anti-human multiclonal BCL-2 antibody or Rabbit anti-human multiclonal BAD antibody were applied. Biotinylated secondary antibody and avidin-biotin-complex with horseradish peroxidase were applied, followed by the addition of the chromogen. Finally, slides were counterstained with hematoxylin, dehydrated in ascending ethanol, cleared with xylene, and mounted with coverslips using a permanent mounting medium. Result: According to the percentage of the dyeing positive cells(A), The dyeing positive cell number of zero is 0, <30% is 1, 30%~60% is 2, >60% is 3. According to the dyeing intensity (B), the achromatic color is 0, the weak dyeing is 1, the dyeing is 2, the strong dyeing is 3; The total score (A + B) ≥ 3 divides into the positive expression, <3 divides into the negative expression. Immunohistochemical results to determine criterion-referenced method of Shimizu [1].

#### 1.2.2 Cell separation, Cell Culture and MTT assay

We adopt mechanical method obtained unicell suspension. First, washed the specimens with normal saline (including penicillin 300 μ/ml streptomycin 300 μ/ml) repeatedly to remove necrotic tissue and blood clots, put in the aseptic plate, then adding them into a little culture medium, used eye scissors cut the specimens into paste, 200 Stainless steel wire grit of 200 mesh screen was cell suspension, it was obtained by filtering the minced tissue, though a stainless steel wire grit of 200 mesh screen, checked for the viability and counted, then centrifuge in 1000 r/min, 10 min; regulated the cell concentration into 5 × 10^4 ^/l by RPMI1640(containing fetal calf serum, penicillin 100 μ/ml streptomycin 100 μ/ml), vaccinated the cell in 96-well microtiter plates,180 μl per well; Each well joined chemotherapeutic agent 20 μl separately (drug level: 10 × PPC, 1 × PPC, 0.1 × PPC), each level set up 3 duplicate holes; Simultaneously set up the cell control group and the blank control group.

Then, the plates were incubated at 37°C in a humidified atmosphere containing 5% CO2 for 48 h. After incubation, 20 μl MTT solution was added to each well, and the plate was incubated for 4 h again; Added DMSO 200 μl, The absorbance of each well was determined in the same manner as used in the MTT assay. The inhibition rate was calculated by using the formula inhibition rate(IR, %) = (1-mean absorbance of the treated wells/mean absorbance of the control wells) × 100%.

### 1.3 Statistical Analysis

The experimental result indicated by the mean ± standard deviation($\bar{x}$ ± S), used the SPSS11.5 statistics software analysis system to carry on the χ^2^-test, and the T-test used for categorized variables and the Spearman rank used for continuous variables correlation analysis. It is considered to be statistically significant difference when P < 0.05.

## Results

### 2.1.1 The expression of BCL-2, BAD in breast carcinoma, breast fibroadenoma and normal breast tissues

**Figure 1:**
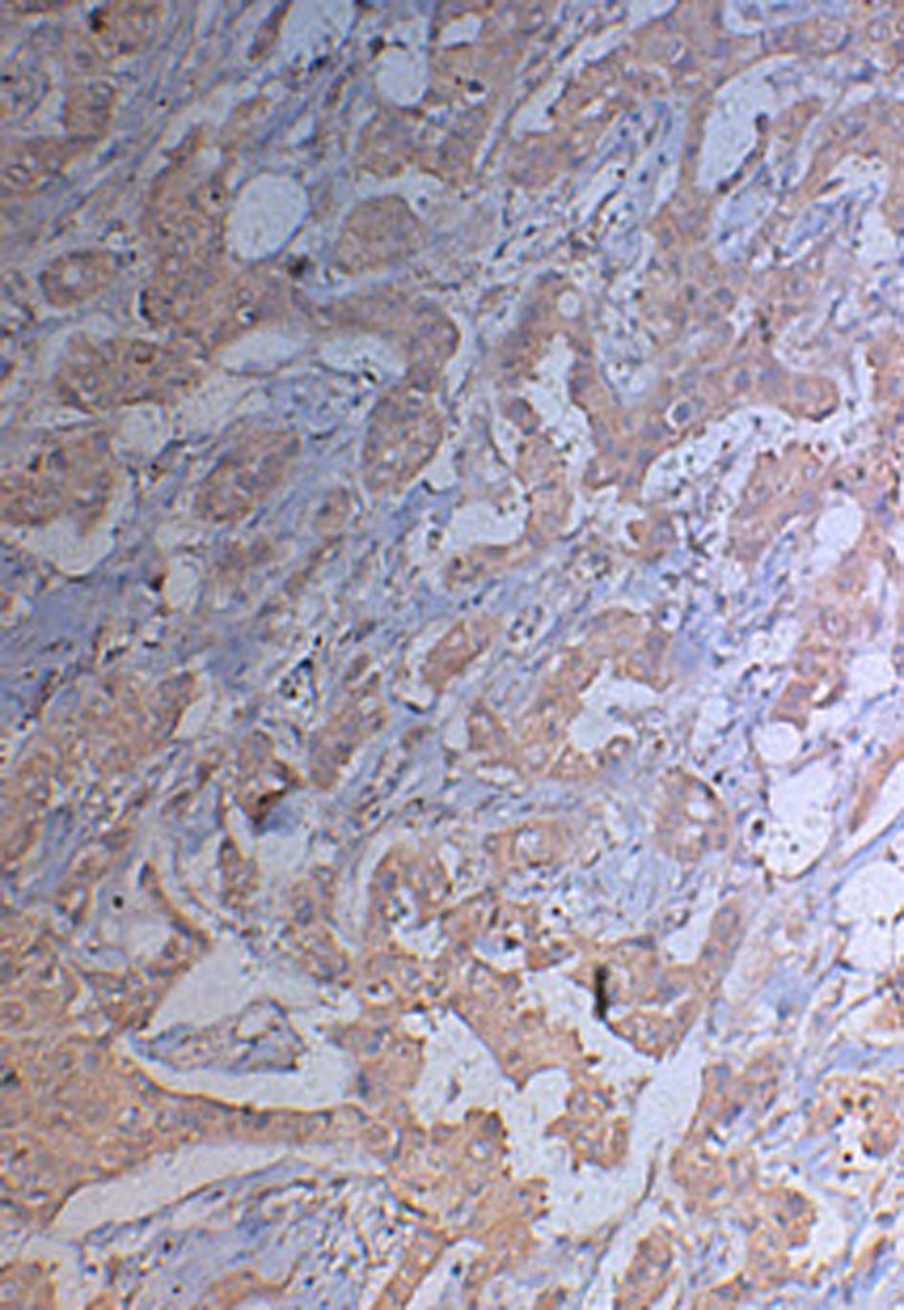
**The positive immunohistochemical expression of BCL-2 in breast carcinoma tissue**.

**Figure 2:**
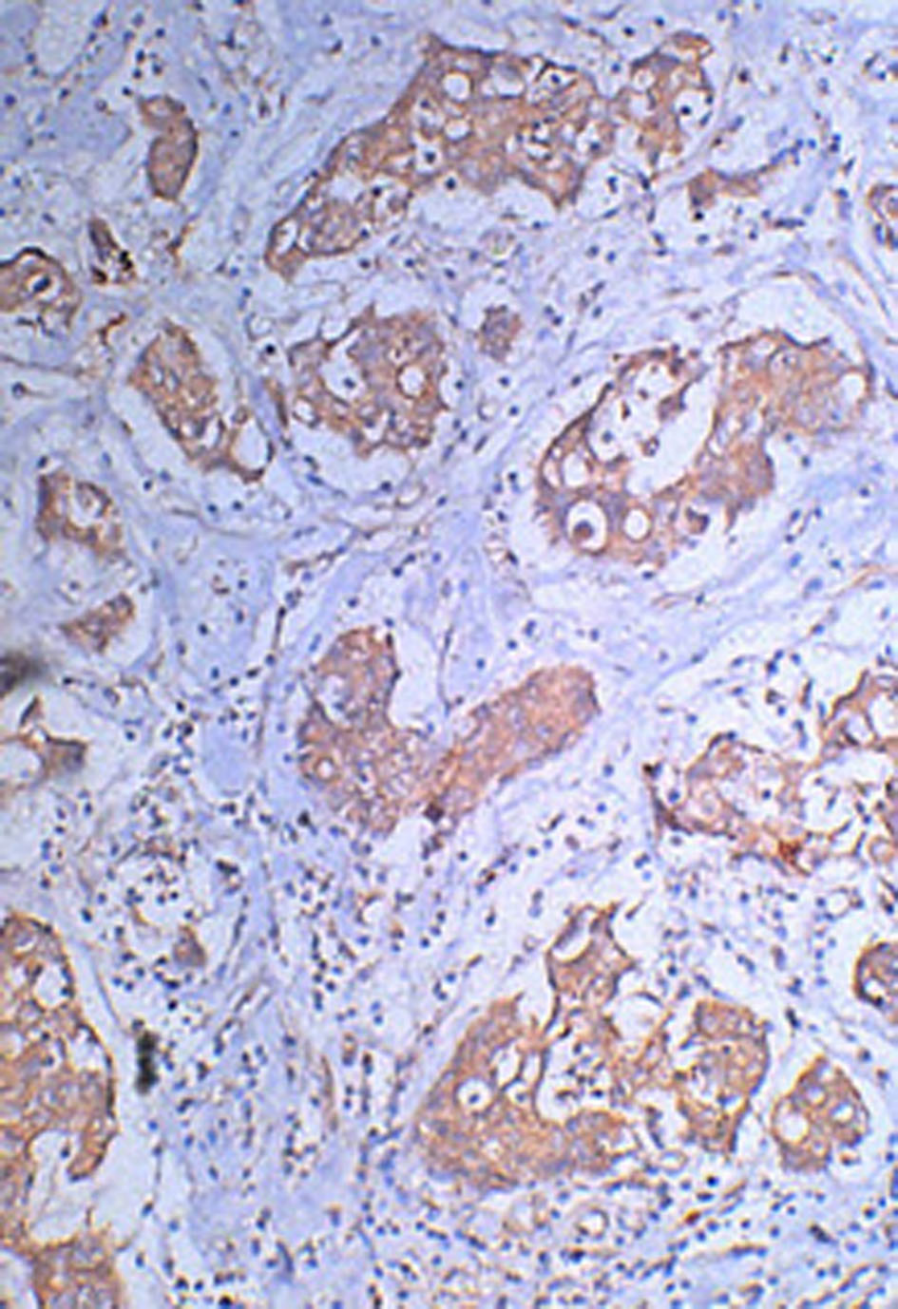
**The positive immunohistochemical expression of BAD in breast carcinoma tissue**.

The expression of BCL-2 and BAD gene in breast carcinoma tissues were indicated by brown granules, mainly distributes in the cytoplasma, and non-uniform; The positive expression of BCL-2 and BAD in breast carcinoma(Fig 1, 2). The expression rates of BCL-2 were normal breast tissue(90%), breast fibroadenoma(80%) and breast carcinoma(61.25%). Compare with the other 2 groups, the expression of BCL-2 was higher in breast carcinoma group, the differences were statistically significant(P < 0.05) (Table 1).

**Table 1:** The expression of BCL-2, BAD in breast carcinoma, breast fibroadenoma and normal breast tissue

	Total	BCL-2			BAD		
		
		+	-	+(%)	+	-	+(%)
Normal breast tissue	10	9	1	90.00%	8	2	80.00%
Breast fibroadenoma	10	8	2	80.00%	7	3	70.00%
Breast carcinoma	80	49	31	61.25%^a^	38	42	47.50%^b^

Compare with the other two groups a P < 0.05; b P < 0.05

### 2.1.2 The expression of BCL-2, BAD in youth and menopause human breast carcinoma

The expression rates of BCL-2 in youth and menopause human breast carcinoma were 47.5% and 75%(P < 0.05), The expression rates of BAD in youth and menopause human breast carcinoma were 30% and 65%, the differences were statistically significant(P < 0.05) (P < 0.05). (Table 2)

**Table 2:** The expression of BCL-2, BAD in youth and menopause human breast carcinoma

	Total	BCL-2			BAD		
					
		+	-	+(%)	+	-	+(%)
Youth group	40	19	21	47.5%^a^	12	28	30%^b^
menopause group	40	30	10	75%	26	14	65%

The two groups compare with each other: a *P *< 0.05 b *P *< 0.05

### 2.1.3 The relationship between the expression of BCL-2, BAD and the histologic grade, clinical stage and the lymph node metastasis in human breast carcinoma

The positive rates of BCL-2 in breast carcinoma histologic grade I, II, III respectively were 84.61%, 58.69%, 12.50%, the difference have stastistical significance(P < 0.01), The positive rates of BCL-2 in breast cancer histologic grades I, II, III to assume the declining trend, statistical analysis showed no significant difference (P = NS). In youth breast cancer histologic grade I, II, III the positive rates of BCL-2 were 87.5%, 44.4%, 0.0%(P < 0.01), and the rates of BAD were 50.0%, 29.6%, 0.0% (P = NS). In menopause breast cancer tissues histologic grade I to III the positive rates of BCL-2 were 88.9%, 73.7%, 0.0%, and the rates of BAD were 61.1%, 68.4%, 33.3% statistical analysis both showed no significant difference, (P = NS).

The positive rates of BCL-2 and BAD were all showed declining trend in the clinical TNM stage from I to IV of youth and menopause breast cancer tissues, but, the difference has no significance (P = NS).

The positive rates of BCL-2 were 15.8% in the youth breast cancer tissues had axillary lymph nodes metastasis, the rates were 76.2% which had no axillary lymph node metastasis(P < 0.01); But the positive rates of BAD showed no relationship with the axillary lymph nodes metastasis. In the menopause breast cancer tissues the positive rates were 20.0% in the axillary lymph nodes metastasis group and 93.3% in control group(P < 0.01); The positive rates of BAD also showed no relationship with the axillary lymph node metastasis in menopause breast cancer tissues(P = NS) (Table 3).

**Table 3:** The relationship between the expression of BCL-2, BAD and the histologic grade, clinical TNM stages and the axillary lymph nodes metastasis in youth and menopause breast cancer tissues

	Total	Histologic grade			Clinical TNM stage				Axillary lymph nodes	
		
		I	II	III	I	II	III	IV	Positive	Negative
Youth breast cancer tissues	40	8	27	5	6	25	8	1	19	21
BCL-2+	19	7	12	0	4	12	3	0	3	16
BCL-2-	21	1	15	5	2	13	5	1	16	5
+%	47.5%	87.5%^1^	44.4%	0.0%	66.7%^3^	48.0%	37.5%	0.0%	15.8%^4^	76..2%
BAD+	12	4	8	0	2	8	2	0	6	6
BAD-	28	4	19	5	4	17	6	1	13	15
+%	30.0%	50.0%^2^	29.6%	0.0%	33.3%^3^	32.0%	25.0%	0.0%	31.6%^5^	28.6%
Menopause breast cancer tissues	40	18	19	3	5	22	11	2	10	30
BCL-2+	30	16	14	0	4	17	8	1	2	28
BCL-2-	10	2	5	3	1	5	3	1	8	2
+%	75.0%	88.9%^2^	73.7%	0.0%	80.0%^3^	77.3%	72.7%	50.0%	20.0%^4^	93.3%
BAD+	25	11	13	1	4	15	6	0	5	20
BAD-	15	7	6	2	1	7	5	2	5	10
+%	62.5%	61.1%^2^	68.4%	33.3%	80.0%^3^	68.2%	54.5%	0.0%	50.0%^5^	66.7%

Compare with each other in the same group:1: *P *< 0.01,2: *P *> 0.05,3: *P *> 0.05,4: *P *< 0.01,5: *P *> 0.05

### 2.1.4 The relationship between the expression of BCL-2, BAD and the expression of ER, PR

All the breast cancer tissues in this study, 9 tissues with the expression of BCL-2 and BAD were positive;In this 9 tissues ER(+)PR(+) of 6 cases(66.7%), ER(+)PR(-) of 2 cases(22.2%), ER(-)PR(+) of 1 case(11.0%), ER(-)PR(-) was 0, When ER(+)PR(+) the positive co-expression rates of BCL-2 and BAD were significantly higher than the other three groups, there were significant differences (P < 0.05). In the 40 cases tissues with BCL-2(+)BAD(-) 18 cases were ER(+)PR(+)(45.0%),10 case were ER(+)PR(-) (25.0%),7 cases were ER(-)PR(+),5 cases were ER(-)PR(-), the difference had no significant difference(P = NS). There were 22 cases with BCL-2(-)BAD(+), which including ER(+)PR(+) 6 cases(27.3%), ER(+)PR(-) 7 cases(31.8%), ER(-)PR(+) 8 cases(36.4%), ER(-)PR(+) 1 case(4.5%), the difference also had no significance(P = NS). There were 9 cases which the co-expression of BCL-2 and BAD were negative, ER(+)PR(-) was 1 case(11.1%),

ER(-)PR(-) were 8 cases(88.9%), ER(+)PR(+) and ER(-)PR(+) were all 0;The negative co-expression rates of BCL-2 and BAD in the ER(-)PR(-) group were significantly higher than the other three groups (P < 0.05).(Table 4)

**Table 4:** The relationship between the expression of BCL-2, BAD and the expression of ER, PR.

	Total	ER(+)PR(+)	ER(+)PR(-)	ER(-)PR(+)	ER(-)PR(-)
Bcl-2(+)Bad(+)	9	6(66.7%)^a^	2(22.2%)^b^	1(11.0%)^c^	0(0.0%)^d^
Bcl-2(+)Bad(-)	40	18(45.0%)	10(25.0%)	7 (17.5%)	5 (12.5%)
Bcl-2(-)Bad(+)	22	6(27.3%)	7(31.8%)	8(36.4%)	1(4.5%)
Bcl-2(-)Bad(-)	9	0(0.0%)	1(11.1%)^f^	0(0.0%)^g^	8(88.9%)^h^

a compared b.c.d P < 0.05; h comparede.f.g P < 0.05.

### 2.2.1 The Sensitivity Of Breast Cancer Cells To Anticancer Drugs In Vitro

The mean relative inhibition rate of breast cancer cells are EADM(69.74 ± 7.67)%, 5-Fu(61.81 ± 9.94)%, NVB(69.10 ± 8.27)%, DDP(63.27 ± 6.79)% in 10 × PPC. The numerus are EADM(45.39 ± 11.74)%, 5-Fu(44.56 ± 12.28)%, NVB(48.50 ± 9.96)%, DDP(41.42 ± 4.81)% in 1 × PPC and EADM(27.57 ± 8.94)%, 5-Fu(25.48 ± 8.62)%, NVB(30.35 ± 9.02)%, DDP(25.33 ± 5.65)% in 0.1 × PPC. Along with drug concentrating reduction, breast cancer cancer cell's inhibition rate relatively reduces gradually. The sensitivity of breast cancer cells to the 4 kinds of drugs in 0.1 × PPC are as follow EADM 30%, 5-Fu 20%, NVB 45%, DDP 25%(Table. 5).

**Table 5:** Sensitivity rate of 20 breast cancer cells to 4 kinds anticancer drugs in 0.1 × PPC

Drugs	Desensitize(%)	Sensitive(%)	Midrange sensitive (%)	Sensitivity rate(%)
EADM	70 (14)	30 (6)	0	30 (6)
5-Fu	80 (16)	20 (4)	0	20 (4)
NVB	55 (11)	35 (7)	10 (2)	45 (9)
DDP	75 (15)	25 (5)	0	25 (5)

### 2.2.2 The Relationship Between The Expression Of BCL-2, BAD And The Chemosensitivity Of The Breast Cancer Cells In 0.1 × PPC In Vitro

In the drug sensitivity test in vitro of breast cancer cells of 4 kinds of chemotherapeutic agents in 0.1 × PPC, the chemosensitivity and the expression level of BCL-2 are related, the chemosensitivity of the BCL-2(-) tumor cells was higher than the BCL-2(+) tumor cells(Table. 6), and there was a negative correlation between the the expression of BCL-2 and the chemosensitivity of the 4 drugs (P < 0.05). In the test the sensitivity to EADM and NVB were associated with the expression of BAD, The BAD(+)tumour cells were more sensitivity to EADM and NVB than the BAD(-)ones(*P *< 0.05)(Table. 7). and there was a positive correlation between the the expression of BAD and the chemosensitivity to EADM and NVB. In the tumour cells which were BCL-2(-)BAD(+) the chemosensitivity to the 4 drugs were higher than the BCL-2(+)BAD(+)and BCL-2(+)BAD(-)ones. The breast cancer cells in which BCL-2 and BAD were all positive were more chemosensitive to NVB than the BCL-2(+)BAD(-)ones(*P *< 0.05); In the tumour cells which were BCL-2(-)BAD(+), BCL-2(+)BAD(-)the chemosensitivity to NVB was higher than to the other 3 drugs, but there were no significant differences(P = NS)(Table. 8).

**Table 6:** The relationship between the expression of BCL-2 in breast cancer cells and the relative inhibition ratio of 4 kinds of anticancer drugs

Drugs	BCL-2			
	
	+	-	*t*	*P*
EADM	30.45 ± 2.52	34.87 ± 2.25	3.99	0.001
5-Fu	30.44 ± 1.49	34.40 ± 2.34	t' = 4.25	0.001^※^
NVB	34.72 ± 3.44	41.19 ± 2.60	4.51	<0.05
DDP	24.32 ± 3.29	29.87 ± 1.90	4.30	<0.05

※*T' *-test

**Table 7:** The relationship between the expression of BAD in breast cancer cells and the relative inhibition ratio of 4 kinds of anticancer drugs

Drugs	BAD			
	
	+	-	*T*	*P*
EADM	39.95 ± 2.29	28.34 ± 6.67	T' = 5.78	<0.05^※^
5-Fu	30.33 ± 3.90	25.76 ± 4.94	1.998	0.061
NVB	38.60 ± 2.67	26.79 ± 6.42	T' = 5.67	<0.05^※^
DDP	28.70 ± 2.56	26.40 ± 2.44	2.044	0.056

※*T' *-test

**Table 8:** The relationship between the combined expression of BCL-2 and BAD in breast cancer cells and the relative inhibition ratio of 4 kinds of anticancer drugs

Drugs	BCL-2(+)BAD(-)	BCL-2(+)BAD(+)	BCL-2(-)BAD(+)	BCL-2(-)BAD(-)
	(***n ***= 8)	(***n ***= 5)	(***n ***= 6)	(***n ***= 1)

EADM	25.93 ± 3.05	33.47 ± 4.65	40.16 ± 5.20	37.72
5-Fu	24.18 ± 4.18	30.38 ± 4.81	37.86 ± 2.80	35.11
NVB	26.06 ± 7.43	36.62 ± 2.78	42.50 ± 2.63	38.88
DDP	23.01 ± 4.14	26.01 ± 4.73	31.90 ± 6.81	28.52

## Discussion

BCL-2 is a gene of anti-apoptosis, the mechanism is possibly related to affect Ca2+ entering the cell, thereby regulating the signal transduction in the cells[2]. BAD and BCL-2 are all members of BCL-2 gene family, and the role of BAD is to promote apoptosis, BAD genes induced apoptosis through to form heterodimers with BCL-2, thus inhibited the anti-apoptotic role of BCL-2 [3] The researches on gastrointestinal tumors, and kidney tumors have found that high expression of BCL-2 of inhibitor of apoptosis, induced tumor growth accelerated, the poor prognosis and poor response to treatment [4,5]. In this study we find that the expression of BCL-2, BAD in tissues of breast carcinoma are significantly lower than tissues of normal breast and tissues of breast fibroma.

Compared with menopause breast carcinoma, youth breast carcinoma shows higher malignant degree, the invasion is stronger, the transfer rate is higher, the prognosis is worse [6]. In this study we found that the expression rates of BCL-2 and BAD in tissues of youth breast carcinoma were significantly lower than in the tissues of menopause breast carcinoma. In breast cancer histologic grade I to III the expression of BCL-2 assumed the decreasing tendency, the differences had significant difference, the expresses of BAD during this process also gradually reduced. The expression of BCL-2 in breast cancer tissues with axillary lymph node metastasis were significantly lower than that without lymph node metastasis. The expression of BAD in the tissues of breast carcinoma and axillary lymph node metastasis showed no correlation obviously, the expression of BCL-2 and BAD were no significant difference in the clinical TNM staging of breast carcinoma. The above data showed that the expression of the two genes played an important role in the occurrence and development of the breast carcinoma; and the changes of BCL-2 and BAD occured in the early stage of the breast carcinoma.

This study showed that the expression of BCL-2 and the expression of ER and PR were highly correlated. In the ER and PR-positive groups, the expression of BCL-2 was significantly higher than that of its negative group;But the expression of BAD showed no significant correlation with the expression of ER and PR. Compare with the BCL-2 negative group, the expression Of ER and PR were higher in the BCL-2 positive group. When the expression of BCL-2 and BAD were positive, at the same time the expression of ER and PR were especially high. Milella [7]. also confirmed that the expression of BCL-2 was regulated by estrogen. The expression of BCL-2 most confined to the ER-positive breast cancer cells, ER-positive was a necessary condition in endocrine therapy; the patient with BCL-2 high expression having a good prognosis, maybe more sensitivity to endocrine therapy. The expression of BCL-2 and BAD can be used as prognosis factors of breast cancer. Detecting the expression of the BCL-2 protein expression level, in particular the combined detection of the expression of BCL-2 and BAD as well as ER and PR were helpful in the prognosis of breast carcinoma.

Chemotherapy is an important treatment means of breast cancer. When we choose chemotherapeutic agents in clinical there still have certain blindness. By using the same chemotherapy, the curative effect of different individuals have large difference. If we did not know the sensitivity of chemotherapeutic agents and utilized them blindly, there would be a lot of side effects. To avoid the side effects we needed to understand the sensitivity of chemotherapeutic agents before the chemotherapy start, and let the treatment individualization. Therefore, before chemotherapy the drug sensitivity, to forecast it becomes necessary, especially. Most chemotherapeutic agents killed tumor cells through inducing apoptosis, thus to investigate the regulatory factor in the procession of apoptosis will provide us a insight to know mechanism of the drug resistance.

BCL-2 and the members of this family plays an important role in regulating the process of cell apoptosis. BCL-2 is the anti-apoptotic gene, its mechanism is not yet clear, and it may affect Ca^2 + ^into cells, thereby regulating cell signal transduction, disturbing the adversed function of free radical and so on [8]. The expression product of BAD can formed heterodimer with the expression product of the anti-apoptotic members of BCL-2 gene family, thereby reversed the function of them. There was clinical research indicating that the expression level of BCL-2 has positive correlation with some factors which to benefit the prognosis of breast cancer such as progesterone receptor and estrogen receptor, and has negative correlation with the disadvantage ones such as EGFR and the number of metastatic lymph node [9]. In the study of breast disease also found in the procession (normal - simple hyperplasia - atypical hyperplasia - carcinoma in situ - invasive breast cancer) the expression of BAD had a decreasing trend [10].

In this study we found that the sensitivity of the breast cancer cells to the 4 drugs were higher in the BCL-2 expression negative ones. Through the rank correlation analysis we found that there was a negative correlation between the BCL-2 expression and the chemosensitivity in breast cancer, indicated that BCL-2 maybe made the breast cancer cells resistant to chemotherapy drugs through its anti-apoptotic function. BCL-2 possibly became one of the effect prognosis factors to determine the curative effect of the chemotherapy in treatment. In our study the breast cancer cells with BAD expression positive were more sensitivity to EADM and NVB than the negative ones. In the tumour cells which BCL-2(-)BAD(+) the chemosensitivity to the 4 drugs are higher than the BCL-2(+)BAD(+)and BCL-2(+)BAD(-)ones. The breast cancer cells in which BCL-2 and BAD are all positive are more chemosensitive to NVB than the BCL-2(+)BAD(-)ones(*P *< 0.05). We indicated that the union examination of BCL-2 and Bad might play a guiding role in the selection of chemotherapy drugs. Studies [11] confirmed that antisense oligonucleotide of BCL-2 can effectively reduce the expression of BCL-2 in breast cancer cells, reduced the inhibition caused by the BCL-2 gene in chemotherapy-induced apoptosis, improved the treatment effect. Antisense oligonucleotide of BCL-2 as an enhancer of the chemotherapeutic effect, provided us a new way for the treatment of breast cancer.

## Competing interests

The authors declare that they have no competing interests.

## Authors' contributions

BY did the immunohistochemical assay, XS: preformed the statistical analysis, and participated in culturing cell in vitro, HYS culturing the cell in vitro, FG did the MTT test, YMF colleted the sample, ZJS designed the experiment, analysed the data, and wrote this paper. All authors read and approved the final manuscript.

## References

[B1] Shimizu M Saitoh Y Itoh H Immunohistochemical staining of Ha-ras oncogene product in normal, benign, and malignant human pancreatic tissues [J] Hum Pathol 1990 21 6 607 612 10.1016/S0046-8177(96)90006-4 2161789

[B2] Mounia Chami Andrea Prandini Bcl-2 and Bax Exert Opposing Effects on Ca2+ Signaling, Which Do Not Depend on Their Putative Pore-forming Region [J] Biol Chem 2004 279 52 54581 54589 10.1074/jbc.M409663200 15485871

[B3] Datta SR Katsov A Hu L Petros A Fesik SW Yaffe MB Greenberg ME 14-3-3 proteins and survival kinases cooperate to inactivate BAD by BH3 domain phosphorylation [J] Mol Cell 2000 6 41 51 10.1016/S1097-2765(00)00006-X 10949026

[B4] Kim R Emi M Therapeutic potential of antisense Bcl-2 as a chemosensitizer for patients with gastric carcinoma Journal of Clinical Oncology 2005 23 16S(June 1 Supplement) 4050

[B5] Molto Luis Rayman Pat The Bcl-2 Transgene Protects T Cells from Renal Cell Carcinoma-mediated Apoptosis Clinical Cancer Research 2003 9 4060 4068 14519627

[B6] Agrup M Stal O Olsen K Wingren S C-erbB-2 overexpression and survival in early onset breast cancer [J] Breast Cancer Res Treat 2000 63 1 23 29 10.1023/A:1006498721508 11079156

[B7] Milella M Trisciuoglio Daniela Bruno Tiziana Trastuzumab Down-Regulates Bcl-2 Expression and Potentiates Apoptosis Induction by Bcl-2/Bcl-XL Bispecific Antisense Oligonucleotides in HER-2 Gene-Amplified Breast Cancer Cells [J] Clinical Cancer Research 2004 10 7747 7756 10.1158/1078-0432.CCR-04-0908 15570009

[B8] Chami M Prandini A Campanella M Pinton P Szabadkai G Reed JC Rizzuto R Bcl-2 and Bax exert opposing effect on Ca^2+ ^signaling, which do not depend on their putative pore-forming region [J] J Biol Chem 2004 279 52 54581 10.1074/jbc.M409663200 15485871

[B9] Linjawi A Kontogiannea M Halwani F Edwardes M Meterissian S Prognostic significance of p53, Bcl-2, and Bax expression in early breast cancer [J] Am Coll Surg 2004 198 1 83 10.1016/j.jamcollsurg.2003.08.008 14698315

[B10] Xiao-wei Wang Bing-lin Guo Zhong-hua Shang Bcl-2 and Bad protein expression in breast carcinoma [J] Chin J Gen Surg 2004 19 9 564

[B11] Tanabe K Kim R Inoue H Emi M Uchida Y Toge T Ant3. isense Bcl-2 and HER-2 oligonucleotide treatment of breast cancer cells enhances their sensitivity to anticancer drugs [J] Int J Oncol 2003 22 875 81 12632082

